# *Nebria brevicollis* (Fabricius, 1792) in North America, benign or malign? (Coleoptera, Carabidae, Nebriini)

**DOI:** 10.3897/zookeys.147.2119

**Published:** 2011-11-16

**Authors:** James R. LaBonte

**Affiliations:** 1 Oregon Department of Agriculture, Plant Division, 635 Capitol Street N.E., Salem, OR 97301-2532, U.S.A.

**Keywords:** *Nebria brevicollis*, invasive species, Carabidae, North America, Oregon, Washington

## Abstract

*Nebria brevicollis* (Fabricius) is one of the most frequently encountered and widely distributed carabid beetles in Europe. Until recently, the only North American records were based on two single specimens, both from the 1930’s in southeastern Canada. In 2008, this species was found at thirteen different sites in five counties in northwestern Oregon. As of the end of 2010, it has been found in thirty-four different sites in ten Oregon counties, with a north-south range of ~150 km and an east-west range of ~90 km. It was also detected in 2010 in southwestern Washington (Vancouver), just north of Portland and the Columbia River.

The ecological amplitude of *Nebria brevicollis* in Oregon rivals that of the most eurytopic native carabid species, e.g., *Pterostichus algidus* LeConte and *Scaphinotus marginatus* (Fischer von Waldheim). It has been found in highly degraded heavy industrial sites, agricultural fields, city parks, gardens, second growth woodlands, mature conifer forests, montane rock gardens, and otherwise pristine stands of old growth noble fir, with elevations ranging from essentially sea level to 1,249 meters. Climates at these locales vary from that of the Mediterranean Willamette Valley floor, where snow rarely occurs and summers are hot and dry, to the summit of the Oregon Coast Range, where deep snow may be present from November through April and summers are cool. The carabid communities in which *Nebria brevicollis* has been found range from those predominantly of fellow exotic species, e.g., at heavily perturbed sites, to those where it is the only exotic species, such as at the Coast Range summit.

*Nebria brevicollis* is clearly an invasive species in that it is not restricted to anthropogenic habitats, is rapidly expanding its North American range, and can be abundant in essentially pristine settings. What is not yet clear is whether it is or will become a damaging species. Although it is already the most abundant carabid species in some settings, based upon pitfall catches, it is unknown whether this represents competitive superiority, trap vulnerability, or utilization of previously untapped or non-limiting resources. Deleterious ecological effects could include not only competition with other predators (including other carabid species) in agricultural and natural settings but also predation upon non-adult stages of threatened and endangered species of butterflies.

## Introduction

There are approximately sixty species of exotic Carabidae established in North America (Y. Bousquet in prep, Bousquet and Larochelle 1993, P.M. Hammond in litt., Kavanaugh and LaBonte 2008, Spence and Spence 1988). The vast majority of these species are almost exclusively synanthropic in the sense of Spence (1990) in that a species is both common in and more-or-less restricted to disturbed, anthropogenic habitats. A few of these species may be confined to natural habitats imbedded in natural or anthropogenic settings, e.g., *Metacolpodes buchanani* Hope is arboreal on shrubs and trees (Togashi 1993) and *Trechoblemus westcottii* Barr is apparently subcortical (J.R. LaBonte, unpublished data). Several other exotic species are specialists in habitats subject to frequent disturbance, such as some anthropogenic habitats as well as riparian areas, e.g., *Elaphropus parvulus* (Dejean) (LaBonte and Nelson 1998) and marine beaches above the average high tide zone, e.g., *Harpalus affinis* (Schrank) (J.R. LaBonte, unpublished data). Two exotic carabid species could be described as habitat generalists successful in both anthropogenic habitats and relatively pristine habitats, *Carabus nemoralis* O.F. Müller and *Pterostichus melanarius* (Illiger). *Carabus nemoralis* has been found to be abundant in conifer forests at moderate (E. van den Berghe in litt.) and high (J.R. LaBonte, unpublished data) elevations in Idaho and in mesic mixed coniferous/broadleafed forest in Maine (R.E. Nelson, unpublished data), while *Pterostichus melanarius* has been found in subalpine meadows in Oregon (J.R. LaBonte, unpublished data) and various forested habitats in Alberta, Maine, and Québec (Cardenas and Buddle 2007, R.E. Nelson, unpublished data; Niemalä and Spence 1994; Spence this volume, Spence and Spence 1988). *Carabus granulatus* Linnaeus, reported from old fields and red alder stands in Canada (Spence and Spence 1988), may meet the criteria of a habitat generalist as well, but I don’t know of it from undisturbed habitats in the Pacific Northwest.

Research on exotic species of carabids in North America to this point indicates that most appear to be ecologically neutral or benign, having little, if any, deleterious or beneficial effects on native carabids, on other biotic elements, or on native biota in native habitats. This is largely a consequence of the strong synanthropic associations of most of the exotic carabids, which are perceived as filling and being more-or-less resticted to a largely empty set of anthropogenic niches otherwise occupied by indigenous species of carabids typical of open habitat or by a few indigenous habitat generalists (Spence 1990, Spence and Spence 1988). As mentioned previously, incursion of *Carabus nemoralis* and *Pterostichus melanarius*, as well as *Carabus granulatus*, into less disturbed and sometimes forested habitats is somewhat counter to this generalization, at least in terms of restriction to anthropogenic habitats. Targeted research revealed no substantial effects of *Pterostichus melanarius* on native forest carabid species (Niemalä and Spence 1991, 1994). As reported in Kavanaugh and LaBonte (2008), *Carabus nemoralis* was found to be much more abundant than the indigenous *Carabus taedatus* Fabricius at several forested locales in Idaho. However, this information stemmed from casual, short term observations rather than prolonged surveys and there were no previous data on the abundance of the native species prior to the introduction of the exotic species.

**Figure 1.**
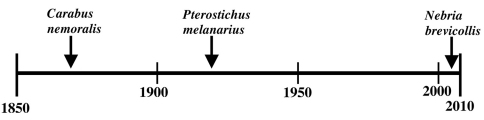
Approximate timeline of detections of *Carabus nemoralis*, *Pterostichus melanarius*, and *Nebria brevicollis* in North America.

Given their presence in non-anthropogenic habitats, can either *Carabus nemoralis* or *Pterostichus melanarius* be considered invasive species rather than relatively benign, more-or-less ecologically neutral additions to our fauna? There are a great many definitions of invasive species. However, a practical definition I favor is that an exotic species is invasive if it establishes and reproduces in natural, relatively undisturbed, non-anthropogenic habitats as well as those of human origins AND also has detrimental effects on the native biota OR has deleterious economic (including detrimental effects on desirable exotic biota) or human health effects. Based on this definition, it seems that neither species meets the preceding definition of invasive exotic species. Although both species have been found in and are presumably reproducing in relatively undisturbed forest habitats, to this point neither appear to have detrimental effects on the native carabid species or other native organisms in those habitats. Both species have been associated with reduced native carabid species diversity in anthropogenic habitats but this was not felt to be a pronounced overall effect even in those situations (Spence and Spence 1988). Considering that *Carabus nemoralis* has been in North America since at least 1870 (Lindroth 1961–1969, p. 37) and *Pterostichus melanarius* since at least the mid-1920’s (Brown 1950) (Fig. 1), their invasive capabilities seem to be weak at most.

**Figure 2.**
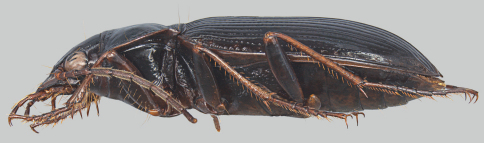
Lateral habitus of *Nebria brevicollis*.

**Figure 3.**
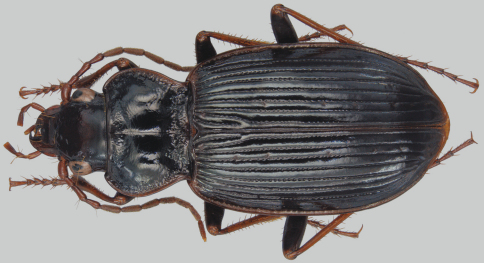
Dorsal habitus of *Nebria brevicollis*.

*Nebria brevicollis* (Fabricius) (Figs 2, 3) was found to be established in western Oregon in 2008, with the earliest specimens extending only back to 2007 (Kavanaugh and LaBonte 2008) (Fig. 1). At that time, the extent of the known distribution was about 100 km north to south from Portland to Corvallis and about 50 km east to west from Troutdale to Dallas, at thirteen more-or-less geographically distinct sites in five counties. Other than the Dallas record, the known sites were in the center of the northern Willamette Valley and all were at low elevations of 165 m or less. Habitats were generally amid heavily industrialized and disturbed urban areas but also included intensively managed blueberry and strawberry fields. Not surprisingly, the carabid faunas of these sites were dominated by exotic species and several eurytopic indigenes. The exception to these strongly disturbed sites was at Dallas, where the habitat consisted of lightly disturbed, more-or-less closed canopy mature second growth mixed conifer/deciduous forest. Other than *Nebria brevicollis*, the carabid species collected at this site were all indigenous.

Given the habitat breadth demonstrated by *Nebria brevicollis* in Oregon, the question was raised in Kavanaugh and LaBonte (2008) whether this recently detected exotic species would compete with indigenous forest species to their detriment. Placed in a larger context, the question is whether *Nebria brevicollis* is invasive in North America, in contrast to the apparent ecological impacts of most of the remaining fifty-nine or so exotic species of carabids known from this continent. Does it meet the criteria of an invasive species? In other words, is *Nebria brevicollis* benign or malign in North America?

## Is Nebria brevicollis rapidly expanding its range in North America?

**Figure 4.**
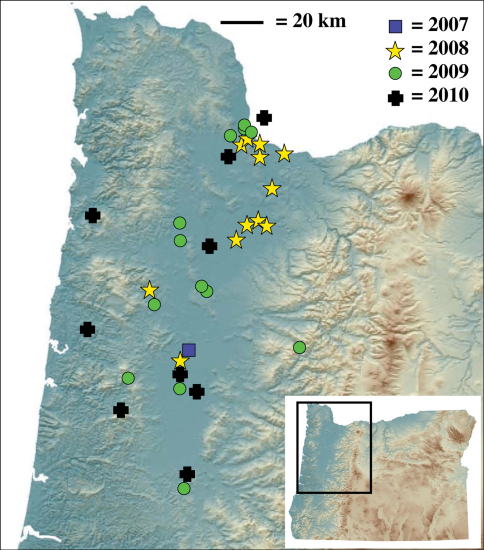
Known localities of *Nebria brevicollis* in North America through 2010 and first year of detection per site.

Although not strictly part of my definition of an invasive species, rapid range expansion is certainly characteristic of invasive species (e.g., Elton 1958). It appears that *Nebria brevicollis* is rapidly expanding its range in western North America. The original detections in 2008 found this beetle in approximately thirteen distinct sites in five counties, covering an area roughly 100 km north to south by 50 km east to west. As of the end of 2010, *Nebria brevicollis* has been found in thirty-four distinct sites in Oregon and ten Oregon counties, twenty-one more sites and five more counties than in 2008. As predicted in Kavanaugh and LaBonte (2008), it has now been found in Washington state: WA, Clark County, Vancouver, Riveridge Park, 45.6053°N, 122.5459°W, 17 April – 2 May 2010, pitfall, A. Karankou collector (A. Karankou and P. Messer in litt.). The total known range now covers an area approximately 150 km north to south and 90 km east to west (Fig. 4), an increase north to south of 50 km and east to west of 40 km.

While rate of detection is not necessarily equivalent to rate of range expansion, there is strong evidence for rapid range expansion of this exotic species following recent introduction into Oregon (Kavanaugh and LaBonte 2008). Additionally, as noted therein, the earliest record of *Nebria brevicollis* in Oregon is based on only a single 2007 specimen from Corvallis, despite relatively intense local insect collecting for research and student entomology class collections at Oregon State University for well more than the past fifty years. In 2010, this beetle was represented in more than half the student collections (D.R. Maddison pers. comm.), indicative of rapid population increase and expansion into suitable local habitats, which is associated with similar phenomena on a larger scale. As discussed below, *Nebria brevicollis* has also become extremely abundant in most of the habitats in which it is present, many of which have been well sampled by local entomologists in the past, further evidence of rapid infilling of available local habitats. That expansion has been rapid rather than gradual with a longer presence in Oregon is also supported by finding *Nebria brevicollis* in abundance at sites where it was not found by recent collecting. For example, I have extensively collected Marys Peak at such sites via pit falls 1984–1985 and via manual collection in 2003 (and sporadic, frequent collecting between and after those periods), as well as the summit of Prairie Peak in 1993. The distribution of this beetle within the periphery of its known range in Oregon is not yet continuous, as would be expected if it had been present for a prolonged period. For instance, although it is abundant at the summit of Prairie Peak, it is apparently absent (as demonstrated by intensive collecting) from suitable habitat 2 km to the north and 200 m lower in elevation.

This rapid range expansion has been fueled by both the pronounced eurytopy of *Nebria brevicollis* and effective mechanisms of dispersal. Members of this species in our region not only have flight capability but indeed disperse in this manner. All specimens I’ve seen of *Nebria brevicollis* in Oregon have fully developed wings and I have found numerous individuals in Lindgren flight intercept funnel traps. Furthermore, it is likely that these insects have been, and continue to be, dispersed over long distances through anthropogenic pathways such as firewood, sod, miscellaneous yard debris, potted plants, and hitchhiking in or on vehicles. Dispersal into remote areas may have been via the latter means. Such stratified dispersal, combining short range diffusive dispersal with satellite populations established far ahead of the main expansion front via anthropogenic means and subsequently coalescing with core populations unless separated by unsuitable habitat, is typical of exotic invasive insect species (Liebhold and Tobin 2008).

## Does Nebria brevicollis establish and reproduce in native, non-anthropogenic habitats?

**Figure 5.**
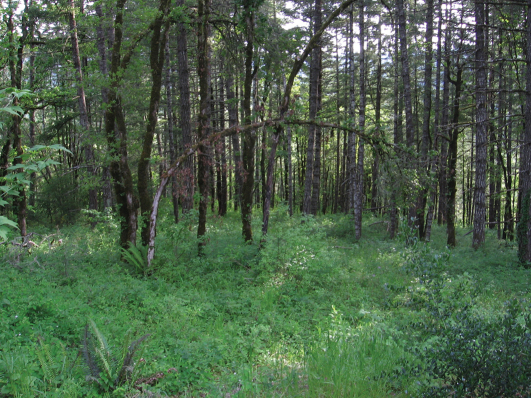
Second-growth mixed forest near Dallas, Oregon.

**Figure 6.**
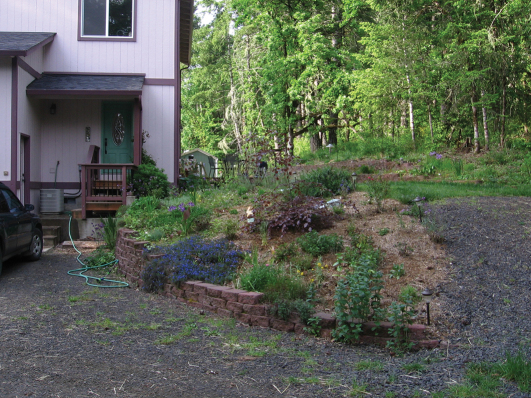
Rural homesite amid second-growth mixed forest near Dallas, Oregon.

Kavanaugh and LaBonte (2008) found *Nebria brevicollis* flourishing in at least lightly disturbed mixed forest at low elevation (Fig. 5). Nonetheless, it could be legitimately argued that this was not a natural and non-anthropogenic setting as the area had undergone rural residential development with homes (Fig. 6) and agricultural endeavors imbedded therein and the forest had been cut some decades previously. In this manner, *Nebria brevicollis* was behaving in a manner similar to that documented for *Carabus nemoralis* and *Pterostichus melanarius*. However, since Kavanaugh and LaBonte (2008), *Nebria brevicollis* has been found to be abundant in several natural and non-anthropogenic habitats. In at least two instances, these habitats were virtually pristine.

**Figures 7–10.**
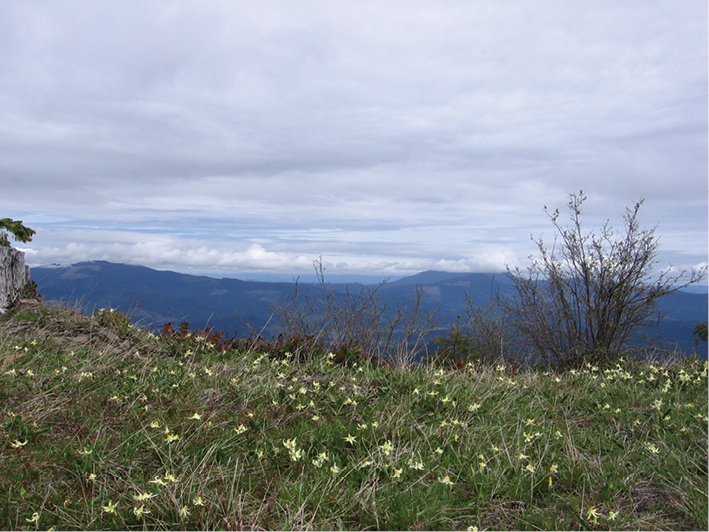
Oregon Coast Range, Prairie Peak **7** (top left), view of Oregon Coast Range to north from summit **8** (top right), grass bald at summit **9** (bottom left), glacier lily (*Erythronium oregonum*) patch at summit **10** (lower right), rock garden at summit..

Sites outside of the eastern and western margins of the Willamette Valley (Fig. 4) are amid non-anthropogenic and largely natural habitats. The easternmost site is at a remote locale in the western foothills of the Cascade Mountains at an elevation of 1,220 m and consists of old growth coniferous closed canopy forest of noble fir (*Abies procera*), western hemlock (*Tsuga heterophylla*), and Douglas-fir (*Pseudotsuga menziesii*). The three westernmost locales in which *Nebria brevicollis* was found in 2010 are all in forested areas in the Coast Range. One of these, Prairie Peak, is quite remote and accessible only by a dirt road and is amid mature second-growth Douglas-fir and western hemlock forest. Nonetheless, *Nebria brevicollis* was found to be very abundant in the essentially undisturbed native grass bald habitat (Figs 7–10) at 1,040 m elevation, even via casual collecting.

**Figures 11–14.**
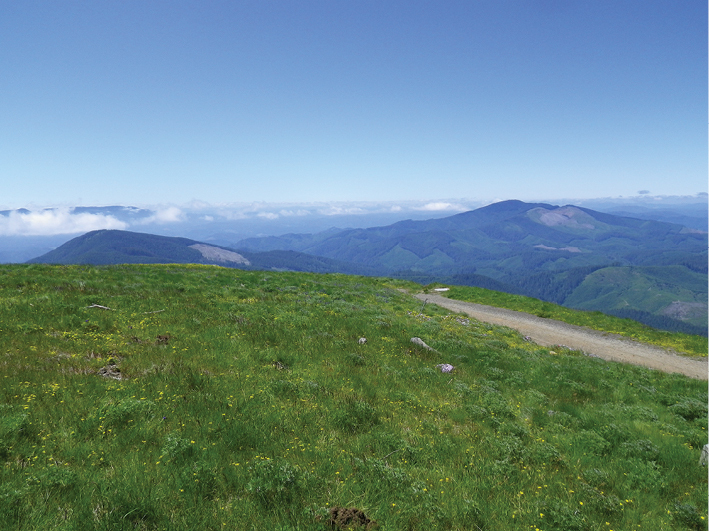
Oregon Coast Range, Marys Peak **11** (upper left), Marys Peak in May, seen from the Willamette Valley south of Corvallis **12** (upper right), meadow at summit (courtesy D.R. Maddison) **13** (lower left), old growth noble fir stand on north flank **14** (lower right), old growth noble fir stand on northwest flank.

Perhaps the most disturbing locale, in an ecological context, where *Nebria brevicollis* has been found in abundance is at and below the summit of Marys Peak. This mountain is at the crest of the Oregon Coast Range, at an elevation of 1,250 m (Fig. 11). Higher elevations on the peak often remain snow covered from mid-November through March or April. An extensive grass bald and meadow system extends throughout the subsummit and the vicinity of the summit (Fig. 12). A large rock garden is present among the exposed bedrock at the summit. A forest dominated by mature and old growth noble fir with some western hemlock and Douglas-fir is present from near the summit down to about 900 m (Figs 13 and 14). Most of the stands are closed canopy, although numerous gaps are present. This forest has not been logged, although most of the trees date from a stand-replacing fire in the late 1840’s. Although a few exotic species of carabids can be found in disturbed areas along the access road, manual collecting and a 1984-1985 pitfall study have demonstrated that the forest carabid fauna is pristine, consisting only of indigenous species. In the pitfall study, twenty-one species were found, with eight species comprising ~96.5% of all individuals captured (J.R. LaBonte, unpublished data).

In September 2009, I was appalled to find large numbers of *Nebria brevicollis* under cover in the closed canopy stands along the north flank of Marys Peak (Figs 13 and 14). This was at one of the sites I had pitfalled in 1984–1985 and I had frequently collected there since that time. Subsequent collecting found *Nebria brevicollis* to be extremely abundant throughout forest, meadow, and summit areas. In many situations, it was the most common carabid found. During one collecting trip in late June 2010, it was the only abundant carabid to be found at the summit in the rock garden and adjacent meadows. Turning rocks often revealed a dozen or more individual *Nebria brevicollis*.

In summary, *Nebria brevicollis* exhibits tremendous ecological amplitude in Oregon, being found from virtually at sea level to over 1,200 m in elevation and is abundant in habitats ranging from desolate industrial sites to pristine montane old growth forest. Such ecological range is unrivaled by any other exotic carabid species in Oregon and is only matched by two extremely eurytopic indigenous species, *Pterostichus algidus* LeConte and *Scaphinotus marginatus* (Fischer).

## Is Nebria brevicollis a competitor of other carabid species?

Exotic species newly present in a habitat can cause populations of indigenes or previously established exotics to decline through competition for limiting resources such as food or shelter, via predation, or by introduction of novel parasites and pathogens (e.g., Crowder and Snyder 2010). With regard to whether *Nebria brevicollis* meets the definition of ‘invasive’, one question is whether it causes populations of indigenous carabid species to decline in its presence. It could also be regarded as invasive if it has deleterious effects on previously established exotic carabid species which provide beneficial services to humans via arthropod or weed biocontrol. Unfortunately, determining whether resident carabid populations are declining when sympatric with *Nebria brevicollis* has proven to be difficult.

There is no question that *Nebria brevicollis* is extremely abundant in some Oregon habitats. Such hyper-abundance is typical of invasive insect generalist predators, which often reach densities several orders of magnitude greater than similar indigenous species (Crowder and Snyder 2010). Unpublished data by J.C. Lee from pitfall surveys of ground-associated predators and pests in blueberry fields showed *Nebria brevicollis* to comprise 21% (N=210) of the carabid fauna in a Benton County field and 54.1% (N=5,443) of the carabid fauna in a Clackamas County field. The other numerically dominant species in the Benton County field were *Pterostichus algidus* (51.9%) and *Pterostichus melanarius* (15.7%), with the remaining ten species of carabids, mostly indigenes, comprising the remaining 11.1%. Other than *Nebria brevicollis*, the only numerically dominant carabid species in the Clackamas County field was *Pterostichus melanarius* (21.8%), with the remaining 24.1% comprised of twenty-five species, mostly exotics. Pitfall data from surveys for exotic plant pests conducted by the Oregon Department of Agriculture throughout Oregon found *Nebria brevicollis* to be very abundant in many of the areas of the Willamette Valley surveyed. For instance, eighty-seven *Nebria brevicollis* were found in a single pitfall over a two-week period at the margins of Forest Park, a largely natural forested park at the northwestern boundary of Portland. This species has even been elevated to the status of occasional home invader where in one instance hundreds of *Nebria brevicollis* were found in the crawl space of a residence in Salem. As mentioned previously, it is now one of the most readily encountered species among the otherwise virtually pristine carabid fauna of Marys Peak. However, high abundance of this exotic species does not necessary equate to deleterious effects on resident species of carabids.

If *Nebria brevicollis* is indeed causing other carabid species to decline in its presence, there are several mechanisms through which deleterious impacts could be effected, including competitive displacement (for resources or via interference) or intraguild predation (Snyder and Crowder 2010). Given the modest size of final instar larvae and adults of this insect, it seems unlikely that intraguild predation upon other adult carabids by *Nebria brevicolllis* would be significant, except perhaps upon substantially smaller species, such as members of *Bembidion* Latreille or *Trechus* Clairville. Predation by immature and adult *Nebria brevicollis* upon non-imagines of other carabid species seems much more likely to have substantive effects thereon, but the frequency of this behavior is unknown. Interference competition by *Nebria brevicollis* upon other carabid taxa is certainly possible, but has not been documented.

The most probable means by which *Nebria brevicollis* would negatively affect populations of other carabid species would be via competition for food or shelter. Displacement of ecologically similar indigenous species by introduced invasive insect generalist predators occurs with striking regularity (Snyder and Evans 2006) and ecologically equivalent sympatric exotic species would seem equally vulnerable. Given the modest size of most carabid species sharing habitats with *Nebria brevicollis* in Oregon and the nature of those habitats, I think it is unlikely that shelter is a limiting resource.

Competition would seem most likely to be greatest for food (presuming that it is not in unlimited supply) and most intense among those species with diets and phenologies most similar to that of *Nebria brevicollis*. Although there appears to be surprisingly little data available on the diet of *Nebria brevicollis*, it appears to fit the archetypal carabid profile as an opportunistic predator taking a wide array of prey within the limitations imposed by morphology, phenology, habitat, and behavior. *Nebria brevicollis* has been documented as feeding on molluscs (e.g., slugs), earthworms, mites, Opiliones, spiders, Collembola, and the eggs, larvae, pupae, and adults of various small insects, including Coleoptera (e.g., Elateridae), Diptera (e.g., Anthomyiidae), and Lepidoptera (small caterpillars) (Larochelle 1990, Mair and Port 2001). Examination of the crops of a few individuals from Oregon revealed that ants are also eaten (J.R. LaBonte unpublished data).

Although there is rather little published feeding data on the indigenous species found in Oregon with *Nebria brevicollis*, it appears that most are opportunistic predators or omnivores and the same is true for the exotic species (e.g., Larochelle 1990). Total body length is an important constraint on the size of prey non-specialized predatory carabids can successfully subdue and feed upon. *Nebria brevicollis* is a rather modest-sized carabid, ranging in length from about 10–14 mm (Lindroth 1961–1969) and Penney (1966) noted that most of the prey taken by *Nebria brevicollis* were less than 4 mm in length. A laboratory study on prey size selection by several European species of *Pterostichus* Bonelli (Wheater 1988) supports the possibility of competition for food between *Nebria brevicollis* and *Pterostichus algidus* (and other opportunistically predatory carabids of similar dimensions). The total length of *Pterostichus algidus* ranges from about 10–16 mm (Hatch 1953, Lindroth 1961–1969). Wheater found that 90% of prey taken by *Pterostichus nigrita* (Schaller) (average total length 10.8 mm) were 5 mm or less in length and 68% of prey taken by *Pterostichus madidus* (Fabricius) (average total length 14.6 mm) were in that size range. Even the substantially larger *Pterostichus melanarius* (average total length 16.5 mm) selected 50% of prey at sizes 5 mm or less. Thus, the size of prey taken by *Nebria brevicollis* is within the prey size preferences of even those species of *Pterostichus* of greater average length.

**Figures 15–16.**
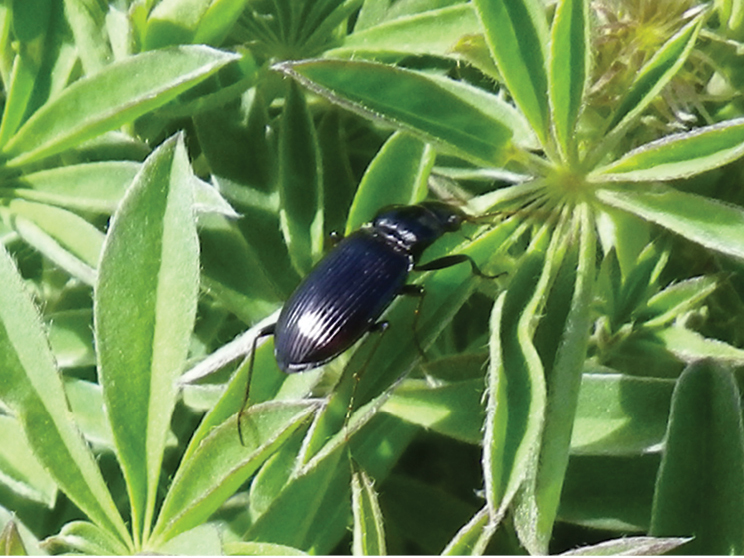
Oregon Coast Range, Marys Peak, rock garden meadow at summit. Images courtesy of D.R. Maddison, **15** *Nebria brevicollis* on grass stems, **16** *Nebria brevicollis* on lupine vegetation.

The capabilities for opportunistic predation of *Nebria brevicollis* are apparently greater than those of other *Nebria*, for this species has been observed foraging over grasses (Fig. 15) and forbs (e.g., lupine, Fig. 16) in mid-day on Marys Peak (J.R. LaBonte and D.R. Maddision, personal observations). This behavior has not been formerly documented for *Nebria brevicollis*, let alone for any other species of *Nebria* (D.H. Kavanaugh, pers. comm.). Numerous individuals at several locales were observed behaving in this manner. The possibility that vegetation was used as “launch pads” for dispersal flights was considered, but no flight or pre-flight behavior was observed. The beetles were traveling up and down grass stalks and wandering over lupine leaves and were clearly engaged in investigative and foraging behavior. Access to non-prostrate vegetation expands the potential prey resources for *Nebria brevicollis* beyond the epigean habitats to which many other predatory carabids are restricted. Combined with the marked eurytopy of this beetle, such foraging behavior may enable *Nebria brevicollis* to achieve higher population densities and subsequent competitive advantage over sympatric carabid species (e.g., Crowder and Snyder 2010, Polis et al. 1997).

A final question in this regard is whether the phenology of *Nebria brevicollis* in North America differs sufficiently from sympatric carabids to provide temporal refugia from competition for those species. For instance, several studies have concluded that spring-active native carabid species can coexist with the predominantly summer active *Pterostichus melanarius* (Hatten et al. 2007, Niemela et al. 1997), suggesting that sympatric carabid species with phenological histories similar to those of *Nebria brevicollis* would be most vulnerable to competition. In Britain, *Nebria brevicollis* had a distinct summer diapause, with a sharp decline in adult activity beginning in June and virtually no adult activity in July and August (Penney 1969). Adults became active again in early September and continued activity until temperatures became too cold in winter. A similar pattern has been observed in Oregon (J.R. LaBonte, unpublished data; J.C. Lee in litt.). However, adult activity at higher elevations in Oregon appears to be somewhat different, with many seen actively foraging in late June (J. R. LaBonte and D.R. Maddison, personal observations). Adults have been found running as early as mid-March and as late as mid-November in the Willamette Valley (J.R. LaBonte, unpublished data). It appears that, at least at low and moderate elevations in Oregon, a temporal refugiam from adult *Nebria brevicollis* competition may exist during winter, early spring, and summer. However, *Nebria brevicollis* may not be completely inactive during the winter and early spring since Williams (1959) found evidence of at least sporadic breeding during winter in Britain, based on the presence of first-instar larvae from mid-September through late April. The normally mild winter temperatures of western Oregon at modest elevations could increase the frequency and duration of winter activity in adult *Nebria brevicollis*.

It is important to remember that the phenological patterns discussed above refer to adult phenologies. Larvae of carabid species which have imaginal temporal refugia from interaction with adult *Nebria brevicollis* are still vulnerable to potential competition, not only from adults but from the larvae. For instance, since the larvae of *Nebria brevicollis* are active from fall through spring (Greenslade 1964, Penney 1969, Thiele 1969, Williams 1959), carabids with larvae present during this period are presumably most exposed to negative effects (which could conceivably also include predation). The interactions of *Nebria brevicollis* larvae with other carabid species may be the most significant because the bulk of the population metabolism occurs within this stage, as shown by Manga (1972), who concluded that larval metabolism accounted for 65% of the total energy flux of the population studied.

There is little question that *Nebria brevicollis* has great potential for deleterious effects on sympatric species of other carabids. This exotic species is extraordinarily eurytopic, can attain extremely high populations, is polyphagous, and is active during periods when other carabid species would be vulnerable to competitive interactions and, possibly predation. However, at this time, there is no unequivocal evidence that negative impacts have occurred.

## Does Nebria brevicollis have deleterious effects on other, non-carabid, taxa?

The apparently opportunistic predation of adult *Nebria brevicollis* upon small and modest-sized invertebrates combined with the great abundance achieved by this exotic predator in many habitats could pose threats to some non-carabid taxa, especially some threatened and endangered Lepidoptera. It has been found at Mount Hebo (Tillamook County) (D.R. Maddison, personal observation), one of the few remaining locales of the federally listed threatened Oregon silverspot butterfly, *Speyeria zerene hippolyta* (Edwards). Eggs of this butterfly are laid on low vegetation near host plants, blue violet (*Viola adunca*) and larvae and pupae are on violet foliage (McCorkle and Hammond 1988, Pyle 2002). Since violet foliage is virtually prostrate on the ground, foraging *Nebria brevicollis* would have little difficulty coming into contact with larvae, pupae, or recently eclosed adult silverspots, while eggs are probably vulnerable to climbing *Nebria brevicollis* adults. First-instar silverspot larvae may be particularly vulnerable as they overwinter in duff and the crowns of host plants (J.F. Miller, Oregon State University, pers. comm.; McCorkle and Hammond 1988). Consequently, this life stage is exposed to the potential depredations of the winter-active larvae of *Nebria brevicollis*, as well as any adults that may be active at that time.

*Icaricia icarioides fenderi* (Macy), the Fender’s blue butterfly, is a federally listed endangered subspecies that is also conceivably threatened by *Nebria brevicollis*. During spring and summer, non-adult stages are on the host plant, Kincaid’s lupine (*Lupinus sulphureus kincaidii*) (Pyle 2002). The observations of foraging on lupine foliage by *Nebria brevicollis* raise concerns that, as with Oregon silverspot butterflies, they could prey on eggs, larvae, pupae, or recently eclosed adult Fender’s blues. Second-instar larvae diapause in duff during late summer, fall, and winter (Pyle 2002) and are thus exposed to potential predation by *Nebria brevicollis* larvae and adults during this period. Although I don’t know of any documented presence of *Nebria brevicollis* at specific Fender’s blue locales, the distribution of this butterfly is confined to the Willamette Valley (Pyle 2002), which appears to be the core of the known *Nebria brevicollis* distribution (Fig. 4). This beetle thrives in habitats similar to those where Fender’s blues are found and if it is not yet at those sites, it soon will be.

Whether the occurrence of *Nebria brevicollis* at sites where threatened and endangered butterflies exist constitutes a risk to those insects is unknown. However, these opportunistic predators are known to feed on the eggs, larvae (including caterpillars), and pupae of a wide variety of insects. There is no *a priori* reason to presume that they would refrain from feeding on any available prey of suitable size. Of course, this also raises the question of what other threatened or endangered insects (or those that are potentially so) within the current or future North American range of *Nebria brevicollis* would be threatened by this exotic predator. For instance, the Oregon Biodiversity Information Center (2010) currently lists 102 species and subspecies of insects as being rare, threatened, or endangered. Although many of these taxa are outside of the known distribution of *Nebria brevicollis* or are found in situations that would render them at low risk from this species (for instance, twenty-four species are caddisflies, Trichoptera), some could conceivably be preyed upon by this beetle. Of course, the effects of a hyper-abundant, eurytopic, opportunistic exotic predator on non-threatened species of other insects or invertebrates are even more speculative.

## Conclusions

*Nebria brevicollis* is rapidly expanding its range in western Oregon and Washington. It is also an extraordinarily eurytopic species, demonstrating a habitat breadth surpassing any previously established exotic species of Carabidae in Oregon. Only a few indigenous carabid species rival its eurytopy. Very high populations can be attained in all these habitats. It is also a very polyphagous opportunistic predator within the prey size constraints imposed by morphology. Unlike most carabid species sharing its habitats, *Nebria brevicollis* is also capable of foraging on erect vegetation. These are all earmarks of an exotic species with great potential for invasiveness. While *Nebria brevicollis* clearly meets the criteria of an invasive species with regard to range expansion and incursions into non-anthropogenic habitats, it is not known whether it is having deleterious effects on sympatric carabid species or non-carabid taxa.

This is a golden opportunity to assess the effects of a recently introduced and established exotic species. It is rare that the early stages of invasion are well documented, as in this case. I encourage those colleagues with the interest and resources to investigate this intriguing situation.
